# Novel Treatments and Technologies Applied to the Cure of Neuroblastoma

**DOI:** 10.3390/children8060482

**Published:** 2021-06-07

**Authors:** Irene Paraboschi, Laura Privitera, Gabriela Kramer-Marek, John Anderson, Stefano Giuliani

**Affiliations:** 1 Wellcome/EPSRC Centre for Interventional & Surgical Sciences, University College London, London WC1E 6BT, UK; i.paraboschi@ucl.ac.uk (I.P.); Laura.Privitera@gosh.nhs.uk (L.P.); 2 Preclinical Molecular Imaging, Division of Radiotherapy and Imaging, The Institute of Cancer Research, London SM2 5NG, UK; Gabriela.Kramer-Marek@icr.ac.uk; 3 Cancer Section, Developmental Biology and Cancer Programme, UCL Great Ormond Street Institute of Child Health, London WC1N 1EH, UK; j.anderson@ucl.ac.uk; 4 Department of Specialist Neonatal and Pediatric Surgery, Great Ormond Street Hospital for Children NHS Foundation Trust, London WC1N 3JH, UK

**Keywords:** Neuroblastoma, Monoclonal Antibodies, Antibody-Drug Conjugates-Based Therapy, Third-Generation Tyrosine Kinase Inhibitor, Drug-Loaded Nanoparticles, cellular immunotherapies, tumour vaccines, radiation therapies, intra-operative treatments

## Abstract

Neuroblastoma (NB) is the most common extracranial solid tumour in childhood, accounting for approximately 15% of all cancer-related deaths in the paediatric population1. It is characterised by heterogeneous clinical behaviour in neonates and often adverse outcomes in toddlers. The overall survival of children with high-risk disease is around 40–50% despite the aggressive treatment protocols consisting of intensive chemotherapy, surgery, radiation therapy and hematopoietic stem cell transplantation2,3. There is an ongoing research effort to increase NB’s cellular and molecular biology knowledge to translate essential findings into novel treatment strategies. This review aims to address new therapeutic modalities emerging from preclinical studies offering a unique translational opportunity for NB treatment.

## 1. Introduction

Neuroblastoma (NB) is the most common extracranial solid tumour in childhood, accounting for approximately 15% of all cancer-related deaths in the paediatric population [1]. It is characterised by heterogeneous clinical behaviour in neonates and often adverse outcomes in toddlers.

The overall survival of children with high-risk disease is around 40–50% despite the aggressive treatment protocols consisting of intensive chemotherapy, surgery, radiation therapy, and hematopoietic stem cell transplantation [2,3].

There is an ongoing research effort to increase NB’s cellular and molecular biology knowledge to translate essential findings into novel treatment strategies. This review aims to address new therapeutic modalities emerging from preclinical studies offering a unique translational opportunity for NB treatment.

## 2. Novel Molecules and Nanoparticles

### 2.1. Monoclonal Antibodies (mAbs)

**Figure 1:**
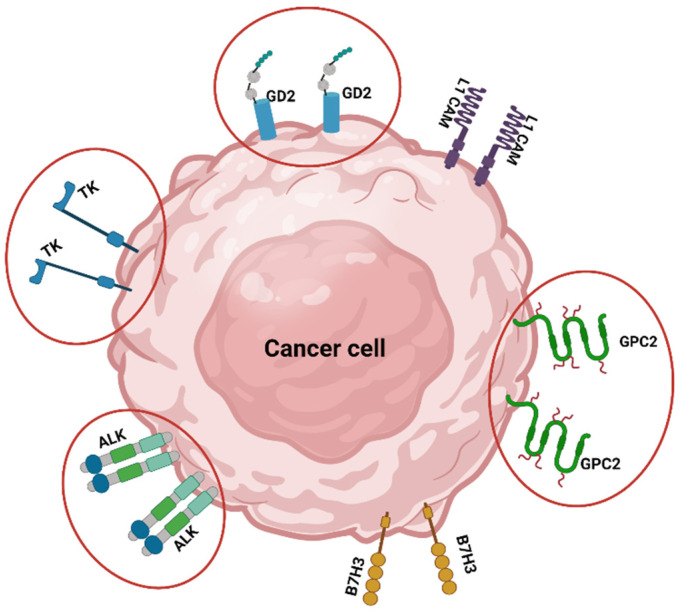
Molecular targets in Neuroblastoma. The image shows 6 different targets: tyrosine kinases (TK); GD2; L1 cell adhesion molecule (L1 CAM); glypican-2 (GPC2); B7H3, and anaplastic lymphoma kinase (ALK). Molecules highlighted in red discussed in paragraph 2.

Specific mAbs against NB-associated antigens have been investigated as the basis for different immunotherapeutic approaches. Several authors have tried to enhance the efficacy of anti-GD2 mAb ch14.18 (dinutuximab and dinutuximab beta), which is now standard of care for patients with high-risk NB in Europe and North America, by combining its administration with immunologically active molecules (Figure 1) [4,5]. The primary mechanism of action of dinutuximab is most commonly presumed to be antibody-dependent cell cytotoxicity (ADCC) mediated by cells such as natural killers (NK), monocytes, macrophages, and neutrophils [6].

The induction of immune checkpoints represents an important mechanism used by tumours to escape immune system recognition and growth. NB cells, for example, upregulate Programmed death-ligand 1 (PD-L1) expressed on effector T cells. PD-1 inhibitory receptors have been adopted in combination with ch14.18/CHO-based immunotherapy in preclinical studies. In vivo experiments showed a significant reduction of tumour growth and prolonged survival when PD-L1+/GD2+ NB-bearing mice were treated with ch14.18/CHO combined with anti-PD-1 mAb [4].

Regulatory T cells (Treg), both natural and peripherally converted, represent a crucial mechanism of tumour-related immunosuppression, and they may limit the onset of an efficient anti-tumour immune response. This phenomenon was studied by Croce et al. [7], who demonstrated that the transient depletion of CD4(+) T cells augmented IL-21-based immunotherapy of disseminated NB in syngeneic mice. Moreover, the combined immunotherapy with anti-PD-1/anti-PD-L1 mAbs and anti-CD4 mAbs resulted in a synergistic effect, leading to a significant increase of tumour-free survival in two syngeneic models of disseminated NB [8]. Another study published by Tran et al. [5] showed that the addition of galunisertib, a TGFβR1 inhibitor, to adoptive cell therapy with NK cells plus anti-GD2 mAb reduced the tumour growth and increased the survival of NSG mice injected with NB cells (Table 1).

### 2.2. Antibody-Drug Conjugates (ADC)s-Based Therapy

Molecularly targeted approaches have been extensively investigated for NB treatment to lower systemic toxicity and improve clinical outcomes. In particular, ADCs are a rapidly growing class of anticancer drugs that, by combining the targeting properties of mAbs with potent cytotoxic drugs, have been emerging as efficient therapies against NB.

Amongst the NB-specific cell surface molecules, glypican-2 (GPC2) can be considered an ideal immunotherapeutic target being overexpressed on high-risk NBs and restricted on normal childhood tissues (Figure 1). This finding was exploited to develop the D3-GPC2-PBD molecule, consisting of an IgG1 anti-GPC2 mAb conjugated to pyrrolobenzodiazepine (PBD) dimers, a class of potent cytotoxic DNA minor groove interstrand crosslinking agents. The D3-GPC2-PBD mediated potent dose-dependent cytotoxicity in an antigen- and concentration-dependent manner in vitro and was similarly efficacious and safe for in vivo NB murine models [9].

Another target for ADCs therapy has been the anaplastic lymphoma kinase (ALK) protein. ALK is widely expressed in most high-risk primary NB tumours regardless of the mutation status, particularly in patients with advanced-stage and MYCN-amplified disease [10]. In detail, a highly specific ALK mAb (CDX-0125) has been conjugated with thienoindole (TEI), a DNA minor groove alkylating agent, to promotes cell death in ALK-expressing NB models [10].

Although initially only tumour-associated antigens were considered ideal targets for ADCs, more recently, the stromal components of the tumour microenvironment (TME) also have been explored as potentially actionable targets. In particular, tumour-derived extracellular vesicles (EVs) have been used as a drug delivery system capable of altering the TME and modulating cancer development. Because the EVs derived by most NB cells are highly enriched by the galectin-3-binding protein (LGALS3BP), a non-internalising LGALS3BP ADC called 1959-sss/DM3 was developed to selectively target NB cell lines. The 1959-sss/DM3 molecule was made by combining an engineered humanised anti-LGALS3BP Ab with DM3, a chemical derivative of maytansine with a cell-killing potency in the picomolar range. Interestingly, preclinical mouse models showed high efficacy in eradicating both orthotopic and metastatic NBs [11] (Table 1).

### 2.3. Third-Generation Tyrosine Kinase Inhibitor (TKi)

Tyrosine kinases (TKs) are a subgroup of the protein kinases family that play a pivotal role in regulating cellular activities, resulting in tumourigenesis and cancer progression in NB [12]. The inhibition of TKs has been investigated as a promising strategy to increase the apoptotic rate in NB cell lines. In this regard, Whittle et al. [13] proved that ponatinib (PON), a multi-targeted tyrosine kinase inhibitor, exerted significant anti-angiogenetic effects in orthotopic xenograft mouse models of human NB, significantly inhibiting their growth and vascularity for in vivo experiments. Moreover, Li et al. [12] found that PON significantly inhibits NB cell proliferation and induces cell apoptosis by blocking FGFR1-activated PI3K/AKT/mTOR and JAK/STAT3 signal pathways.

Because an increased rate of resistance to PON was reported in either preclinical or clinical settings, with autophagy being the primarily responsible mechanism, Corallo et al. [14] investigated its cytoprotective role in NB cells during treatment with PON. Interestingly, their data confirmed a PON-dependent activation of autophagy, both in vitro and in vivo, and highlighted the function of autophagy inhibitors, such as chloroquine, as effective adjuvants for enhancing the cytotoxic effects of current chemotherapy protocols (Table 1).

### 2.4. Drug-Loaded Nanoparticles

**Figure 2:**
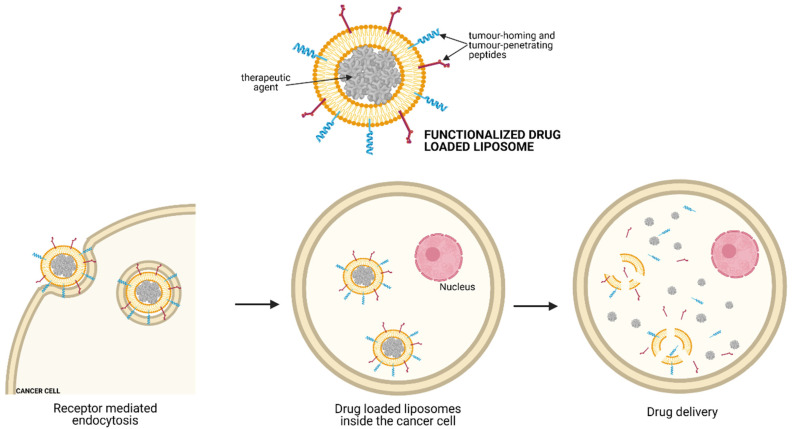
Mechanism of action of drug-loaded liposomes. Liposomes are loaded with anticancer agents and functionalised with peptides capable of recognising the cell of interest. Once the liposome fuses its lipid bilayers with other cell bilayers, the anticancer drugs are released from the liposome into the cancer cells, exhibiting their cytotoxic action.

New approaches based on targeted drug delivery may improve the efficacy and decrease the toxicity of NB treatment. In this respect, innovative therapies based on the use of liposomes loaded with anticancer agents and functionalised with peptides, capable of recognising NB cells and/or tumour-associated endothelial cells, are of particular interest. Because of their composition, liposomes can carry both hydrophilic and hydrophobic molecules. The extent of action of the drug will depend upon its physicochemical characteristics and composition of lipid. The mechanism of drug molecule delivery is based on the fusion of the lipid bilayers with other cell bilayers, such as the cell membrane, to release the liposomal content. As cancer cells consume large amounts of fats to fill the rapid growth requirement, they recognise the liposomes and absorb them. Once the anticancer drugs are released from the liposome into the site, cancer cells are killed by the drug [15]. To optimise the use of these nanoparticles, in addition to the “enhanced permeability and retention effect” related to the leaky vessels typically present in tumours, different strategies have been adopted to overcome the many physiological barriers present in tumour tissues (i.e., enzymatic degradation, phagocytes of the reticuloendothelial system (RES), molecular efflux pumps). In detail, liposomal vectors can be functionalised with tumour-homing and tumour-penetrating peptides, which help anticancer drugs penetrate the tumour, increasing their therapeutic effects (Figure 2) [16].

In the literature, several formulations have been described, for example, containing doxorubicin (DXR), fenretinide (HPR) or bortezomib (BTZ), decorated with peptides capable of recognising receptors in the tumour vasculature and/or in NB cells [16]. DXR-entrapped liposomes have been functionalised with NGR-containing peptides, capable of recognising CD13 expressed by NB endothelial cells, to deliver liposomal DXT to tumour vessels [17]. As a result, their anticancer effects were higher than the untargeted DXR-liposomal formulations, with no systemic toxicity.

The therapeutic potential of novel lipidic nanoparticles entrapping miR-mimics has also been explored to reactivate multiple genes/pathways inhibited in tumour settings. Di Paolo et al. [18] developed an effective nanocarrier for the systemic administration of miR-34a and let-7b, two tumour suppressor (TS) miRNAs depleted in NB tumours. Their findings demonstrated that the targeted liposome administration of miR-34a and let-7b mimics significantly increased the survival of pseudo metastatic NB mice (models able to mimic the typical metastatic spread in advanced stages) and significantly reduced cell proliferation, neo-angiogenesis, and tumour growth in orthotopic models of NB (Table 1).

## 3. Cellular Immunotherapy

Immunotherapies based on immune effector cells, able to recognise tumour-associated antigens and exert specific cytotoxicity against tumour cells, are promising approaches investigated in several preclinical studies. Chimeric antigen receptor (CAR)—modified T-cells are genetically engineered T-cells that express a synthetic immunoreceptor consisting of an antigen-binding ectodomain (e.g., single-chain Fv (scFv)). This directs them to a particular tumour antigen and signalling domains that trigger T-cell activation and proliferation when the foreign antigen is bound. Specifically, NB cells ubiquitously express the GD2 ganglioside, an attractive tumour-associated antigen for cellular immunotherapy.

In 2008, Pule et al. [19] engineered Epstein-Barr virus (EBV)-specific cytotoxic T lymphocytes (CTLs) to express a chimeric antigen receptor directed to GD2 to see if they would receive optimal co-stimulation after engagement of their receptors, enhancing survival and anti-tumour activity. They found that human virus-specific CAR-CTLs persist in higher numbers and for longer times after administration than T cells expressing the same receptor but lacking viral specificity (CAR-ATCs), with tumour regression or necrosis in half of the subjects tested. Following this study, Chrystal U. Louis et al. [20] reported the long term clinical and immunologic consequences of CAR-CTLs and CAR-ATCs in 19 patients with high-risk NB (11 of them being in follow-up for four years from the first study). Their results show that the long-term low-level presence of CAR-expressing T cells is associated with clinical benefits, and that they can induce complete tumour responses in patients with active NB. However, follow-on studies using a third-generation format of the same CAR-T failed to show significant clinical efficacy in patients [21]. Using a different antibody, a phase 1 study by Straathof et al. [22] evaluated 12 children with relapsed or refractory NB treated with escalating doses of second-generation GD2-directed CAR-T cells, increasing intensity of preparative lymphodepletion. Twenty-eight days after the infusion, no patients had an objective clinical response based on the standard radiological response criteria. However, three patients demonstrated regression of soft tissue and bone marrow disease, and two patients experienced grade 2 to 3 cytokine release syndrome.

Bocca et al. [23] further evaluated this by investigating the anti-NB activity of GD2-specific CAR T-cells combined with bevacizumab (BEV), a specific mAb against vascular endothelial growth factor (VEGFR), in an orthotopic xenograft model of human NB. When combined with BEV, GD2-CAR T-cells massively infiltrated the tumour mass and secreted interferon-γ (IFN-γ), which, in turn, upregulated NB cell expression of PD-L1. Concurrently, tumour infiltrating GD2-CAR T-cells expressed PD-1. PD-L1 silencing or blocking strategies were then advocated to enhance the efficacy of such a combination of therapies.

Another attractive cell source for adoptive cancer immunotherapy in NB is represented by gamma delta T lymphocytes (γδ T-cells) due to their innate cytotoxicity. In fact, these cells are able to recognise phosphoantigens, which are natural nonpeptide phosphorylated intermediates of isoprenoid metabolism operating in human cells. Interestingly, tumour cells express one of them at a high level, the isopentenyl pyrophosphate (IPP), especially when exposed to amino bisphosphonates. Thus, tumour cells can be exposed to amino bisphosphonates to let them be recognised and killed by γδ T-cells. Di Carlo et al. [24] showed in preclinical models of NB that the combined treatment with Vδ2+ T-cells (the most common subset of γδ T-cells) and zoledronic acid (ZOL) was able to inhibit tumour cell proliferation and angiogenesis and to induce cell apoptosis, supporting their use as a therapeutic strategy for NB patients. Moreover, Fisher et al. [25] showed that the combination of adoptively transferred Vδ2+ T-cells, expanded in vitro with ZOL and IL-2, with dinutuximab and systemic ZOL suppressed tumour growth compared to antibody or γδT cell-free controls in an immunodeficient mouse model of small established GD2-expressing NB tumours.

Regarding the employment of cytotoxic T lymphocytes (CTL) and NK cells for anti-tumour immunotherapy, one of their limits is represented by the downregulation of HLA-class I molecules by NB cells. However, Spel et al. [26] proved that an increase in NB cell immunogenicity was possible upon their exposure to active NK cells, which sensitise NB cells’ recognition by CTLs. Further evidence of the potential of NK cell immunotherapy as a successful approach in NB treatment was reported by Castriconi et al. [27], who showed that early repeated injections of polyclonal IL-2-activated NK cells significantly increased the survival and reduced the bone marrow infiltration of NB-bearing NOD/SCID mice. Interestingly, low doses of human recombinant IL-2 or IL-15 further enhanced the therapeutic effects.

Driven by the increasing recognition of the pivotal role of tumour vasculature in the survival and growth of solid tumours, there has been a great interest in developing approaches that target and disrupt the existent tumours’ vessels [28,29]. Loi M et al. [30] tagged DXR-loaded liposomes with the CPRECESARSSSRTPSDKY peptide, which targets the aminopeptidase A (APA). The APA is enhanced and active in pericytes associated with tumour blood vessels, and it has been correlated with neoplastic progression [31,32]. What they found in this study is that the combined targeting of both the endothelial and the perivascular cells not only increased the disruption of the endothelial wall but also resulted in a statistically significant enhanced anti-tumour effect [30].

Besides tumour blood vessels, liposomal DXR formulations can also be directed explicitly towards tumour cells. In this regard, NB exposure with DXR-loaded Fab’ fragments of anti-GD2 immunoliposomes in nude mice leads to significantly greater inhibition of cell proliferation (in vitro), and long-term survival rates approaching 100% suggested that total inhibition of the metastatic growth of human NB was happening [33].

Finally, the combined use of liposomal DXR tagged with an NGR-containing peptide, and anti-GD2 mAb has been administered sequentially to target both tumour vessels and cancer cells, obtaining a more significant inhibition of NB tumour growth than each formulation given alone. Apart from DXR, sterically stabilised liposomes loaded with HPR have been developed to improve the encapsulated drug’s therapeutic efficacy, reducing neo-angiogenesis and tumour cell proliferation [18]. Similarly, vascular-targeted-BTZ-loaded liposomal formulations have been employed to effectively inhibit NB growth, minimise side effects, and increase the therapeutic index compared to the free drug [34].

## 4. Tumour Vaccines

The use of tumour cell-based vaccines represents an attractive way of generating anti-NB immunity without increasing the toxicity associated with current radiotherapy and chemotherapy protocols. The vaccines train the immune system to recognise and destroy NB cells after chemotherapy.

In this regard, Bauer et al. [35] designed a multimodal tumour vaccine consisting of irradiated tumour cells infected with the oncolytic IL-12-expressing HSV-1 virus (M002), which produced a stable and specific immunisation in a murine model of intracranial tumour.

Chakrabarti et al. [36] showed that the therapeutic vaccination with neuro-2a cells knock-down for the inhibitor of differentiation protein 2 (Id2- kd) significantly suppressed tumour growth in well-established NB tumours. This anti-tumour effect was even more substantial when combined with checkpoint inhibitors. An increased number of IFN-γ producing CD8+ T-cells and the infiltration of cytotoxic CD8+ T cells within the tumour were responsible for the effect of this novel tumour vaccine strategy.

Moreover, Berger et al. [37] reported that the oral gavage of attenuated Salmonella typhimurium (SL7207), carrying recent generated survivin DNA was able to induce a more robust cellular anti-NB immune response than gene gun application or injection of lentivirally transduced bone marrow-derived dendritic cells (DCs) in a syngeneic mouse model of NB.

Similarly, Fest et al. [38] tested a surviving minigene DNA vaccine (pUS-high) administered using SL7207 as a DNA carrier. It proved that this led to complete NB eradication in over 50% of immunised mice.

Because the first-step enzyme of catecholamine biosynthesis, the human tyrosine hydroxylase (hTH), is an important marker for NB, three DNA vaccine plasmids encoding for human hTHcDNA hTH minigene and hTHcDNA have been administered in combination with IL-12 in syngeneic A/J mice to suppress primary tumour growth and spontaneous metastasis [39].

Gil et al. [40] analysed the ability of therapeutic DC vaccines expressing 47-LDA, a CD166 cross-reactive mimotope of the GD2 ganglioside, to selectively expand adoptively transferred tumour-specific T-cells in lymphodepleted NXS2 NB tumour-bearing syngeneic mice. To deliver the antigenic cassette to the activating Fc gamma receptors, the 47-LDA mimotope was presented to DCs either as a linear polypeptide in conjunction with universal Th epitopes or as a fusion protein with the murine IgG2a Fc fragment (47-LDA-Fcgamma2a). Interestingly, the latter formulation was more effective in the induction of the anti-tumour immune response.

Cheung et al. [41] recently published the results of a phase II trial for a bivalent vaccine with escalating doses of the immunological adjuvant OPT-821, combined with oral β-glucan. The patient cohort was composed of 102 patients with high-risk NB in remission. The words “bivalent vaccine” means that there are two antigens, GD2 and GD3, the carbohydrate antigens prevalent in NB. This vaccine’s rationale is that if the patient can make antibodies against the two antigens in the vaccine, they could also selectively kill NB cells by attracting the patient’s white blood cells to kill the NB. However, these antigens are poorly immunogenic, so in order for the body to make antibodies against them, they link each antigen to a protein called KLH (keyhole limpet hemocyanin) and mix them with a substance called OPT-821. Their results show that the vaccine plus b-glucan elicited robust antibody responses in patients and that higher anti-GD2-IgG1 title was associated with improved survival.

Toll like receptor 9 (TLR9) agonists, such as synthetic oligonucleotides containing unmethylated CpG motifs (CpG ODNs), can be used as vaccine adjuvants because they can directly induce the activation and maturation of plasmacytoid DCs and can enhance the differentiation of B cells into antibody-secreting plasma cells [42]. Brignole et al. [43] evaluated the anti-tumour activity of CpG-containing c-myb antisense oligonucleotides encapsulated with GD2-targeted liposomes in two murine xenograft models of NB. They demonstrated that both the direct inhibition of cell growth, mediated by decreased c-myb protooncogene expression and the indirect CpG-dependent immune stimulation by the NK cell-mediated lysis of tumour cells, resulted in the inhibition of tumour growth, leading to long-term survival in NB-bearing mice. Moreover, as IL-10 is an immune-regulatory cytokine known to suppress macrophages and DC function, the combined administration of CpG ODN-containing liposomes and Abs against IL-10R has proved to prolong immune system activation, leading to better therapeutic results in NB xenografts [44].

## 5. Radiation Therapy

Radiation therapy is an essential component of NB treatment and is typically administered to both the primary tumour bed after surgical resection and metastatic sites after induction chemotherapy. However, even after radiation therapy, the loco-regional relapse in these patients is still high, with approximately 50% of children relapsing and bearing a 5-year survival of only 8% [45].

### 5.1. Proton Beam Therapy (PBT)

As NB patients are very young, undergo intensive multi-agent chemotherapy, and the tumour is often close to radiation-sensitive organs, PBT represents a promising alternative to conventional radiotherapy, especially for reducing the treatment burden associated with it. It is also feasible with very little acute and early late toxicity in the susceptible cohort of very young NB patients.

Different studies on PBT for NB have recently been published, with patients showing similar demographics and treatment strategies before irradiation. In these studies, PBT was performed on the pre-operative tumour bed with 21.6–24 Gray (Gy). In this regard, Hill et al. [46] reported a 5-year local control rate of 97% after a median follow-up time of 48.7 months, while Bagley et al. [47] published a 5-year local control rate of 87% after a median follow-up of 60.2 months.

In a study published this year, Danny Jazmati et al. [48] performed a retrospective analysis of children with high- or intermediate-risk NB who had PBT of the primary tumour site performed during the first-line therapy. Their protocol consisted of PBT doses ranging from 21.0 to 39.6 Gy. In 39 patients, radiation was given to the pre-operative tumour bed with or without an additional boost in case of residual tumour (five patients received PBT to the MIBG-avid residual at the primary tumour site at the time of PBT). Although the patients received total doses above 30 Gy, in line with the previously mentioned studies, they did not observe relevant toxicity and tumour control rates were high, both for the primary site and the metastases.

### 5.2. Near-Infrared Photoimmunotherapy (NIR-PIT)

**Figure 3:**
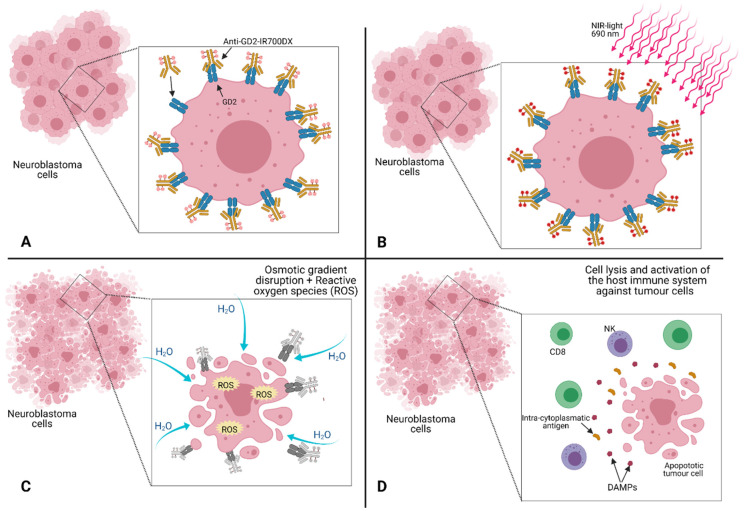
Schematic representation of near-infrared photoimmunotherapy (NIR-PIT) mechanism of action. (**A**) Specific binding of the anti-GD2 monoclonal antibody (mAb) labelled with IRdye700DX (anti-GD2-IR700DX) to the cancer cell surface GD2 antigen (GD2). (**B**) Subsequent local exposure to near infrared (NIR) light. (**C**) The exposure turns on the photochemical “death” switch, resulting in the rapid and highly selective immunogenic cell death (ICD) of targeted cancer cells. (**D**) The rapid cell lysis leads to release of intra-cytoplasmatic antigens and damage associated molecular patterns (DAMPs) in the extracellular space, leading to the activation of the host immune system against the dying tumour cells.

NIR-PIT is a newly developed and highly selective cancer treatment that employs a monoclonal antibody conjugated to a photo-absorber dye (IRDye700DX), which is activated by 690 nm light (Figure 2). It represents a promising anti-tumour strategy capable of enhancing immunotherapy’s therapeutic potential by inducing rapid necrotic/immunogenic cancer cell death. The NIR-PIT, in fact, selectively targets cancer cells and induces anti-tumour host immunity with re-priming and proliferation of T-cells that react against cancer-specific antigens. In particular, NIR-PIT causes direct (Figure 3—panel B) and indirect (Figure 3—panel C) cell killing because the cell membrane rupture releases tumour-specific antigens into the TME and promotes dendritic cell (DC) maturation, resulting in the presentation of cancer-specific antigens on DCs to naive T cells.

At the American Association for Cancer Research meeting in 2018, Hiroshi Nouso et al. [49] presented the use of NIR-PIT with an anti-GD2-IR700 as a promising anti-tumour strategy to enhance the therapeutic efficacy of anti-GD2 immunotherapy for high-risk NB. They evaluated the anti-tumour effect of anti-GD2-IR700 on three human NB cell lines, showing that the administration of anti-GD2-IR700 significantly suppressed the cell viability compared to anti-GD2 mAb when combined with NIR light irradiation. Proceeding further on this line, another study by Yasuhiro Maruoka et al. [50] hypothesised that the administration of IL-15 with cancer cell-targeted NIR-PIT could also inhibit tumour growth by increasing anti-tumour host immunity. To demonstrate this, three syngeneic mouse tumour models underwent combined CD44-targeted NIR-PIT and short-term IL-15 administration. The use of CD44 as a tumour target is a well-known marker of cancer stem cells as it is expressed on the cell membrane of several cancers. Their results show that the combination therapy of IL-15 after NIR-PIT inhibited tumour growth, prolonged survival, and increased tumour infiltrating CD8+ T cells more efficiently than NIR-PIT alone. Thus, IL-15 appears to enhance the therapeutic effect of cancer-targeted NIR-PIT.

### 5.3. Radioisotope Based Radiation

Most NBs express the noradrenaline transporter molecule and take up metaiodobezylguanidine (mIBG), which can be radiolabelled with either ^123^I or ^131^I. The ^131^I-mIBG therapy is currently used for induction and consolidation treatments, with loco-regional control rates of 84–100% in case of persistent MIBG-avid metastatic sites [51].

In 2014 Wilson et al. [52] published a review on ^131^I-mIBG therapy for NB in which they analysed the controversial results present in the literature. Overall, the median response rate of ^131^I-mIBG alone was the same as the radiation combined with chemotherapy. However, response rates reported from different relapsed/refractory studies had a great range of variation. Although the response rates to ^131^I-mIBG therapy were of clinical significance, there was no evidence of better long-term outcome as measured by event-free survival (EFS) or overall survival (OS). Although the analysis was heterogeneous from the clinical perspective, there are still open questions and uncertainties: how effective is ^131^I-mIBG? Should it be administered alone or in combination with chemotherapy?

Enhancing the therapeutic effect of ^131^I-mIBG treatment might require combining it with different treatment modalities. The advantage of combining other radiotherapy modalities lies in the ability to achieve higher radiation absorbed tumour doses without compromising the dose-limiting organs of each therapy. This field was explored by Aurélien Corroyer-Dulmont et al. [53], who improved the molecular radiotherapy outcome through combination with external beam radiotherapy (EBRT) in a mouse model of NB. In their results, vessel permeability was increased at 24 h post EBRT, which correlated with an increase in ^131^I-mIBG uptake. Likewise, EBRT administered 7 days after ^131^I-mIBG significantly decreased the tumour volume and increased overall survival. This study demonstrates the potential of the combined modality EBRT and ^131^I-mIBG therapy as a useful addition to currently available therapeutic protocols.

## 6. Intra-Operative Treatments

### 6.1. Targeted Probes for Surgery

Surgery represents a cornerstone within the multimodal treatment of NB. The literature supports the idea that a gross total resection is associated with better survival outcomes [54,55]. However, performing a radical NB excision is particularly demanding due to the strict adhesion of the tumour to major blood vessels and nerves and the extensive tumour fibrosis resulting from the neoadjuvant chemotherapy. By facilitating the tumour’s discrimination from the surrounding normal tissue, fluorescence-guided surgery (FGS) can lower the risk of intra-operative complications and increase the survival rate of children affected by NB.

In this regard, Wellens et al. [56] developed an anti-GD2-specific tracer consisting of the immunotherapeutic anti-GD2 mAb conjugated to the near-infrared I (NIR-I) fluorescent dye IRDye800CW. Using xenograft mouse models of NB, they proved the specific binding of this fluorescent probe in vivo. They defined an optimal dose of 1 nmol and an imaging time window of 4 days after its administration to obtain a better fluorescent signal.

Similarly, Jin et al. [57] prepared RVG peptide and IRDye800CW-conjugated bovine serum albumin-coated triangular gadolinium oxide nanoplates (RVG&IRDye800-Gd2O3 TNs) both as a targeting MRI agent for the diagnosis of NB and as a fluorescence imaging agent for the guidance of NB surgical removal. Notably, with the guidance of this fluorescent imaging agent, the survival rate of mice bearing orthotopic NB xenografts increased from 0% to 80% 42 days after surgery compared to the survival associated with conventional surgery.

### 6.2. Fibrin Gels (FBGs)

**Figure 4:**
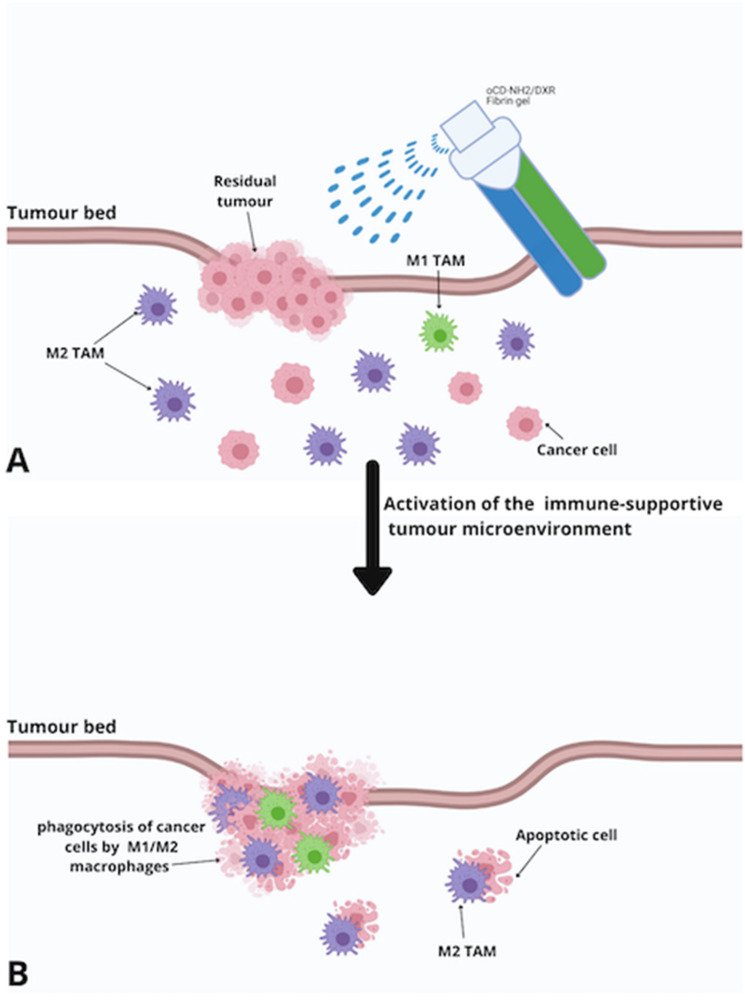
Schematic representation of in-situ-sprayed immunotherapeutic fibrin gel. (**A**) The gel, which contains CaCO_3_ nanoparticles encapsulated with the immunotherapeutic antibody, is sprayed on the tumour bed to be gradually released into the tissue. (**B**) CaCO_3_ nanoparticles scavenge H+ in the surgical wound site, eliciting an immune-supportive tumour microenvironment after surgery. Cyclodextrins (CD) are used to improve the solubility, delivery, and bioavailability of different drugs, such as doxorubicin, leading to higher drug uptake by cells’ antiproliferative and apoptotic activity. Abbreviations: TAM, tumour-associated macrophage. oCD-NH2/DXR, doxorubicin administered in association with functionalised cyclodextrins.

Fibrin gels are widely applied in surgery and are well-known vehicles for the local delivery of anticancer agents. They are highly biocompatible, biodegradable, and safe drug-releasing tools that may overcome the problems related to an insufficient drug concentration at the tumour site. FBG is applied following the surgical resection of the tumour. The gel, which contains CaCO_3_ nanoparticles encapsulated with the immunotherapeutic antibody, is sprayed on the tumour bed to be gradually released into the tissue. The link between the antibody and its target increases the phagocytosis of cancer cells by macrophages and initiates T-cell-mediated anti-tumour response. Furthermore, CaCO_3_ nanoparticles scavenge H^+^ and elicit an immune-supportive microenvironment after surgery [58] (Figure 4).

Vitale et al. [59] evaluated FBGs as a loco-regional therapy in NB with doxorubicin administered in association with functionalised cyclodextrins (oCD-NH2/DXR) in two orthotopic NB models. Cyclodextrins (CD) are used to improve the solubility, delivery, and bioavailability of different drugs, such as doxorubicin, leading to higher drug uptake by cells’ antiproliferative and apoptotic activity. Their results indicated that FBGs loaded with oCD-NH2/DXR had significantly higher anti-tumour activity than the intravenous administration of free DXR applied in the visceral space above the adrenal gland, either pre or post tumour removal. The authors also investigated the marked vesicant and necrotising effects of Dox, limiting its application for loco-regional therapy, by performing histological analysis of different organs at autopsy. Damage related to the application of FBGs loaded with oCD-NH2/Dox was observed in none of the cases, confirming the complete reversibility of sporadic tissue damage observed 6 days after the administration.

The same authors also compared the therapeutic index of FBGs loaded with two effective liposomal doxorubicin formulations (LDFs), Caelyx and Myocet. They evaluated in vivo the local toxicity and anti-tumour activities of FBGs overlaying on the tumour surface, showing that FBGs loaded with Myocet, administered as neoadjuvant or adjuvant treatment, had lower general and local toxicities compared to gels loaded with Caelyx, free DXR, or compared to i.v DXR [60].

## 7. Conclusions

Targeted molecules and nanoparticles, specific cellular immunotherapies, tumour vaccines, radiation therapies, and intra-operative treatments are some of the novel strategies currently under investigation to increase the survival of NB patients. This review aimed to address these new therapeutic modalities, which offer a unique translational opportunity for NB treatment.

## Figures and Tables

**Table 1:** Novel molecules and nanoparticles investigated in preclinical studies focusing on NB treatments.

Author, Year	Title	Investigated Treatment	Results
**Monoclonal Antibodies**			
Siebert N et al. [4], 2017	PD-1 blockade augments anti-NB immune response induced by anti-GD2 antibody ch14.18/CHO	ch14.18/CHO + anti-PD-1 Ab	ch14.18/CHO + anti-PD-1 Ab results in synergistic treatment effects in mice, representing a new effective treatment strategy against GD2-positive NBs.
Croce M et al. [7], 2009	Transient depletion of CD4+ T cells augments IL-21-based immunotherapy of disseminated NB in syngeneic mice	anti-CD4 Ab	Anti-CD4 Ab potentiated IL-21-based immunotherapy by removing Treg cells, their precursors and other CD4+ cell subsets. This allows the development of an IL-21-driven CD8+ Tcell response, which mediates NB rejection.
Rigo V et al. [8], 2017	Combined immunotherapy withanti-PDL-1/PD-1 and anti-CD4 antibodies cure syngeneic disseminated NB	anti-PD-1/PD-L1 Ab	The combined use of anti-PD-1+ anti-CD4 Ab mediated a potent, CD8-dependent, synergistic effect leading to the elongation of mice’s tumour-free survival, complete tumour regression, and durable anti-NB immunity.
Tran et al. [5], 2017	TGFβR1 Blockade with Galunisertib (LY2157299) Enhances Anti-NB Activity of Anti-GD2 Antibody Dinutuximab (ch14.18) with Natural Killer Cells	ch14.18 + TGFβR1 inhibitor (Galunisertib)	Galunisertib suppresses the activation of SMAD2 in NBs and aNK cells, restores NK cytotoxic mechanisms, and increases the efficacy of ch14.18 with aNK cells against NBs.
**Antibody-Drug Conjugates (Adc)S-Based Therapy**			
Bosse KR et al. [9], 2017	Identification of GPC2 as an oncoprotein and candidate immunotherapeutic target in high-risk NB	GPC2 targeting ADC	A GPC2 directed ADC proved to be cytotoxic to GPC2-expressing NB cells in vitro and in vivo.
Sano R et al. [10], 2019	An antibody-drug conjugate directed to the ALK receptor demonstrates efficacy in preclinical models of NB	ALK targeting ADC (CDX-0125-TEI)	CDX-0125-TEI exhibited efficient antigen binding, internalisation and cytotoxicity in cells with different ALK expression. In vivo studies showed that CDX-0125-TEI is effective against ALK wild-type and mutant patient-derived xenograft models.
Capone E et al. [11], 2020	Targeting vesicular LGALS3BP by an antibody-drug conjugate as a novel therapeutic strategy for NB	LGALS3BP targeting ADC (1959-sss/DM3)	LGALS3BP targeting ADC can cure mice with established NB tumours in pseudometastatic, orthotopic and PDX models.
**Third-Generation Tyrosine Kinase Inhibitor (Tki)**			
Li et al. [12], 2017	Novel multiple tyrosine kinase inhibitor ponatinib inhibits bFGF- activated signalling in NB cells and suppresses NB growth in vivo	Ponatinib	Ponatinib can inhibit tumour growth as a single agent or combined with other therapeutic agents, such as doxorubicin.
Whittle et al. [13], 2016	The novel kinase inhibitor ponatinib is an effective anti-angiogenic agent against NB.	Ponatinib	Ponatinib reduces NB cell viability in vitro and reduces tumour growth and vascularity in vivo.
Corallo et al. [14], 2020	Autophagic flux inhibition enhances cytotoxicity of the receptor tyrosine kinase inhibitor ponatinib.	Ponatinib	Inhibition of autophagic flux by CQ restores the cytotoxic potential of PON. In vivo, the use of CQ as adjuvant therapy significantly improves the anti-tumour effects obtained by ponatinib, leading to ulterior reduction of tumour sizes.
**Drug-Loaded Nanoparticles**			
Pastorino F et al. [17], 2008	Enhanced anti-tumour efficacy of clinical-grade vasculature-targeted liposomal doxorubicin.	CD13-targeted liposomal doxorubicin	TVT-DOX proves to be effective in reducing cell proliferation, blood vessel density, and microvessel area, showing increased cell apoptosis.
Di Paolo et al. [18], 2020	Combined Replenishment of miR-34a and let-7b by targeted nanoparticles inhibits tumour growth in NB preclinical models.	GD2-targeted liposomes entrapping miR-34a and let-7b	The replenishment of miR-34a and let-7b by NB-targeted nanoparticles, individually and more powerfully in combination, significantly reduces cell division, proliferation, neoangiogenesis, and tumour growth, induces apoptosis in orthotopic xenografts, and improves mice survival in pseudometastatic models.

Abbreviations. Ab: antibody; NB: neuroblastoma; PD-1: programmed death-1; PD-L1: programmed death-ligand 1; SMAD: small mother against decapentaplegic; aNK: activated natural killer; GPC2: glypican-2; ADC: antibody-drug conjugate; ALK: anaplastic lymphoma kinase; PDX: patient-derived xenograft; CQ: chloroquine; TVT-DOX: Targeted liposomal doxorubicin.
